# Decarceration and COVID-19 infections in U.S. Immigration and Customs Enforcement detention facilities: a simulation modeling study

**DOI:** 10.1016/j.lana.2024.100971

**Published:** 2024-12-27

**Authors:** Christopher Weyant, Jaimie P. Meyer, Daniel Bromberg, Chris Beyrer, Frederick L. Altice, Jeremy D. Goldhaber-Fiebert

**Affiliations:** a Department of Health Policy, Stanford School of Medicine, Stanford, CA, USA; b Center for Health Policy, Freeman Spogli Institute, Stanford University, Stanford, CA, USA; c Section of Infectious Diseases, Yale School of Medicine, New Haven, CT, USA; d Chronic Disease Epidemiology, Yale School of Public Health, New Haven, CT, USA; e Global Health Institute, Duke University, Durham, NC, USA; f Epidemiology of Microbial Diseases, Yale School of Public Health, New Haven, CT, USA

**Keywords:** Incarceration, Immigration detention, COVID-19, Simulation modeling

## Abstract

**Background:**

U.S. Immigration and Customs Enforcement (ICE) facilities had high rates of COVID-19 infections and mortality during the global pandemic. We sought to quantify how many COVID-19 infections could have been averted through different decarceration strategies.

**Methods:**

We developed a set of stochastic simulation models of SARS-CoV-2 transmission in ICE facilities. Employing incremental mixture importance sampling (IMIS), we calibrated them to empirical targets derived from publicly available case time series for ICE facilities, and publicly available facility population censuses prior to vaccine availability (May 6, 2020 to December 31, 2020). The models included infection importation from extra-facility sources. We evaluated reduction of the incarcerated population by 10–90%. People who were decarcerated faced background cumulative risks of infection and detection based on a weighted average of county-level estimates from the covidestim model, which is a Bayesian evidence synthesis model.

**Findings:**

Without decarceration, the infection rate was 5.05 per 1000 person-days (95% CrI 3.40–6.81) and case rate was 1.53 per 1000 person-days (95% CrI 1.04–2.02). Rates declined linearly when decarceration did not reduce contacts of people remaining in facilities and faster than linearly when it did reduce contacts. At all decarceration levels, rates were substantially higher when contacts were not reduced. Even with 90% decarceration, infection rates for people remaining in facilities were higher than or comparable to otherwise similar free-living people.

**Interpretation:**

The decline in COVID-19 infection rates with decarceration was linear or faster than linear depending on how decarceration was implemented. Our findings highlight infection risks associated with incarceration, which compound other health harms of incarceration.

**Funding:**

Stanford’s COVID-19 Emergency Response Fund; the 10.13039/100000026National Institute on Drug Abuse; and the 10.13039/100000025National Institute of Mental Health.

**Research in context**

**Evidence before this study**

We searched PubMed using the following MeSH terms: 1) “decarceration” or “prison release” or “jail release” and 2) “COVID” or “SARS-CoV-2” or “pandemic.” We did not restrict the query to language, location, or dates of publication. The search revealed 145 articles, of which 15 were reviews, 3 were pre-prints, and 10 were commentaries. Adding to the original query an additional search term of “immigration detention” resulted in 10 publications, nearly all of which deployed cross-sectional, qualitative, or retrospective cohort designs. A single prior study (Irvine et al. Journal of Urban Health 2020) modeled the impact of COVID-19 on U.S. Immigration and Customs Enforcement (ICE) detention facilities, but it was not calibrated to actual observed case rates. Adding to the original query an additional search term of “simulation modeling” resulted in 3 publications, none of which investigated decarceration as a public health strategy to reduce infections.

**Added value of this study**

We used publicly available data on actual COVID-19 infections within ICE facilities and the community to model the potential impact of different decarceration strategies in terms of reducing infection rates. This study adds important knowledge on the implementation of decarceration as a public health strategy, estimating the magnitude of health harms that potentially could have been averted had decarceration been optimally deployed.

**Implications of all the available evidence**

Dense carceral settings are associated with serious health risks, including risk of infection with respiratory viruses, as evidenced by the COVID-19 global pandemic. Decarceration strategies, particularily those that include de-densification (reducing the number of contacts for people remaining in facilities), are important for reducing infection risks. However, even with 90% decarceration, infection risks for people remaining in facilities were higher than or comparable to otherwise similar free-living people.

## Introduction

Mass incarceration has resulted in overcrowded and often poorly resourced carceral facilities, generating a “tinderbox scenario” for a droplet- or air-borne respiratory pandemic.^1^^,^^2^ Accordingly, people incarcerated in prisons, jails, and other closed detention settings experienced a COVID-19 case rate over 5-times higher than that of the general population.^3^ COVID-19 risk was especially high for incarcerated populations in the U.S., which was due in part to the U.S. incarcerating more of its citizens per capita than any other country worldwide and having the world’s largest immigration detention system.^4^^,^^5^

Spread of SARS-CoV-2 in carceral facilities posed infection and mortality risks to people within them, as well as to people residing in surrounding communities.^6^^,^^7^ One early county-level estimate was that over half a million COVID-19 cases in summer 2020 (both inside and outside carceral facilities) were attributable to mass incarceration.^8^ Preliminary models also predicted that COVID-19 outbreaks in detention facilities, beyond fueling community spread, would rapidly consume all existing intensive care resources within a 50-mile radius of each facility.^9^ Consequently, it is important for policy makers to consider strategies to mitigate COVID-19 outbreaks in detention facilities.

One mitigation strategy is decarceration, which is a public health and health equity strategy that involves returning incarcerated people and diverting those who would be incarcerated to the community.^10^ Decarceration has been found to reduce or been associated with lower COVID-19 cases in specific carceral settings including a large urban U.S. jail,^11^ Texas prisons,^12^ Massachusetts jails and prisons,^13^ the Allegheny County jail system,^14^ and 101 U.S. non-administrative federal prisons.^15^ Furthermore, studies suggest that decarceration can be implemented in a way that 1) does not increase community crime,^16^ and 2) lowers community case rates.^17^

Based on such evidence, decarceration was recommended to address the COVID-19 pandemic by the National Academies of Sciences, Engineering, and Medicine^10^ and jointly by the World Health Organization, United Nations High Commission on Human Rights, and the Inter-Agency Standing Council.^18^ In contrast, the Centers for Disease Control and Prevention provided guidance on the management of COVID-19 within prisons, jails, and detention settings, but did not make a public statement regarding decarceration.^19^

In most U.S. settings, the size and density of carceral populations were reduced during the public health emergency, not because of more releases, but because arrests and courts ground to a halt, resulting in fewer intakes. In some cases, legal action resulted in decarceration of people who were otherwise close to their release date or transfer of people from facilities to home confinement or furlough. In California prisons, where the incarcerated population fell 19.1% between March 1 and October 10, 2020, a high proportion of people with medical vulnerabilities remained inside facilities and were mostly housed in dormitory settings that were associated with higher COVID-19 infection rates.^20^ Given this variability in how decarceration was implemented across carceral facilities during COVID-19, key questions remain to inform future policy. First, what is the effect of decarceration on COVID-19 cases and further transmission within the U.S. Immigration and Customs Enforcement (ICE) system? Second, how does the level of decarceration influence its benefits? Third, how does the way in which decarceration is implemented (i.e., how contacts change following decarceration) influence its benefits?

We developed simulation models of the ICE system calibrated to retrospective data. We then evaluated how various decarceration strategies, including different levels of decarceration and contact changes, would have altered COVID-19 cases and new infections. The ICE system is a particularly compelling setting to study decarceration because the U.S. system of prisons and jails is otherwise heterogeneous and subject to a wide array of federal, state, and county laws and policies. ICE detains approximately 20,000–50,000 non-citizens per year across 126 facilities who are suspected of committing civil (but not criminal) immigration violations.^21^^,^^22^ Each state has at least one ICE facility and operations may be overseen by ICE, private contractors, or partnering state and local governments or federal agencies. Census and COVID-19 case rate reporting are standardized across all facilities and data is publicly available. We focused our analysis on the pre-vaccine phase of the pandemic as decarceration might be expected to have had a larger health benefit during this phase and there were fewer confounding factors. The aim of the study was to quantify how many COVID-19 infections could have been averted through different decarceration strategies.

## Methods

### Overview

We developed a set of stochastic susceptible (S)-exposed (E)-infectious (I)-detected (D)-recovered (R) (SEIDR) compartmental models of SARS-CoV-2 transmission in ICE facilities.^23^ Employing incremental mixture importance sampling (IMIS), we calibrated the models to targets derived from empirical case time series data and facility population censuses once testing was available and prior to the availability of vaccines (May 6, 2020 to December 31, 2020).^24^, ^25^, ^26^, ^27^ We conducted policy analyses projecting the effects of ICE decarceration at the outset of the pandemic period on cumulative cases and infections during this period. The model was programmed in R version 3.6.3.^28^

### Modeling ICE facilities

**Fig. 1:**
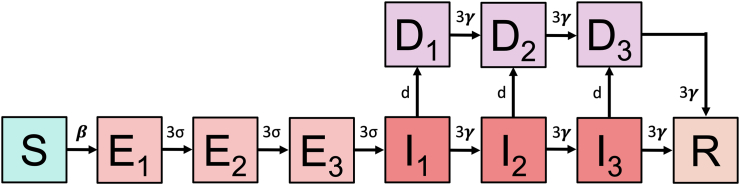
**Model schematic.** We constructed a set of stochastic SEIDR compartmental models of SARS-CoV-2 transmission with one per ICE facility within the ICE system. Shown here is the structure of one such model. There were five health states including: susceptible (S), exposed (E), infected (I), detected (D), and recovered (R). There were three compartments for each of the E, I, and D states to more realistically capture distributions of duration of time spent in each state.

We developed a set of stochastic SEIDR compartmental models of SARS-CoV-2 transmission with one per ICE facility within the ICE system (Fig. 1). All parameters described in this section but not given values were calibrated as discussed in later sections. There were three compartments for each of the exposed (E), infectious (I), and detected (D) health states to realistically capture distributions of duration of time spent in each state.^23^^,^^29^^,^^30^ We modeled population movements between the states. Susceptible people are infected and transition to the exposed state (E) at a rate β, which captures both the contact rate and probability of infection per contact. Exposed people transition to infectious (I) at a rate σ = 0.33 (duration of ∼3.0 days).^31^ Infectious people are detected (D) at rate d and recover (R) (i.e., become no longer infectious) at a rate γ = 0.32 (duration of ∼3.1 days).^31^ Detected people also transition to recovered based on γ. Because detected people may be isolated and have reduced effective contacts, they are less likely to transmit after detection (reduction multiplier π).

We modeled the effects of the pandemic outside of ICE facilities in two ways. First, we allowed infections to be introduced from external sources based on a rate λ. This rate accounts for all possible sources including staff. Second, we allowed for ICE population turnover based on a rate θ. Except for individuals with currently detected infections (D), people could be released from the ICE detention facility; these were randomly selected at rate θ from all other model compartments. These released individuals were replaced by new entrants at the same rate, maintaining the facility’s overall population size; given substantially lower community infection rates during the period of the pandemic we were modeling, new entrants entered the model as susceptible (S).

We assumed that an ICE facility would implement mitigation efforts with some delay ρ after it detects its first case. Representing a variety of interventions, such as distancing (e.g., single-celling) and masking, these mitigation efforts have strength Ω, which reduces the rate of transmission β once they are implemented. We assumed that mitigation effort intensity was reduced to baseline levels after 2 weeks with no detected cases at the facility; mitigation could be reactivated if subsequent cases were detected.

Each ICE facility begins with a population of size N at the start of the model simulation with all individuals susceptible to infection; at some point, the first infection enters the facility and transmission can occur. The simulation follows the facility for 240 days using the tau-leap method with a time-step of 0.1 days; the method is an approximate stochastic simulation method based on Gillespie’s algorithm.^32^

Model outputs included the evolution of the compartment populations over time. They also included: 1) incidence of cases and infections and 2) cumulative cases and infections.

### Calibration targets

We obtained data to calculate calibration targets from a variety of sources (Table 1). We obtained case time series data for ICE facilities over the period prior to vaccine availability (May 6, 2020 to December 31, 2020) from The COVID Prison Project.^26^ We obtained average daily population data for ICE facilities for select dates during this period from TRAC Immigration.^27^ The case time series data was generally available daily while the population data was only available for select dates. Thus, to generate daily population data, we linearly interpolated between the closest available data points for each facility.

**Table 1:** Data sources.

Variables	Data sources
COVID-19 cases in ICE facilities	The COVID Prison Project^26^
Population of ICE facilities	TRAC Immigration^27^
COVID-19 cases and infections in communities	CDC^33^ and covidestim^34^
Population of communities	U.S. Census^35^
COVID-19 generation time	Various^29^^,^^30^^,^^36^, ^37^, ^38^, ^39^

CDC = Centers for Disease Control and Prevention; ICE = U.S. Immigration and Customs Enforcement; TRAC = Transactional Records Access Clearinghouse.

We excluded some of the potential 126 ICE facilities in the data for several reasons (Supplementary Fig. S1). First, we excluded 17 facilities that had case data but no population data (approximately 10% of the facilities with case data) because case rates could not be computed without an at-risk population denominator. Second, we excluded one facility not located in the 50 U.S. states or DC, which was in Puerto Rico. Third, we excluded 13 facilities with a mean population of less than 20 as they accounted for less than 1% of the cases. Fourth, we excluded 17 facilities with less than 200 days of data over the 240-day period; the vast majority of these had less than 100 days of data. After excluding these facilities, 78 facilities remained.

When computing total cases and infections, we rescaled our estimates and uncertainty based on the excluded facilities. For those facilities with less than 200 days of data, we rescaled based on the fraction of population missing. For those facilities with no population data, we rescaled based on the number of missing facilities. We did not account for those facilities with a mean population of less than 20 or not located in the 50 U.S. states or DC as they accounted for less than 1% of the cases.

We calculated model calibration targets from this empirical data. We first calculated targets for the existence and duration of outbreaks. We defined an outbreak as over 2% of the population having detected cases each week for any contiguous period of at least 3 weeks; this threshold roughly corresponds to 5 cases a week in a facility of average size, which was approximately 250 people. We calculated the fraction of facilities that had at least one outbreak and at least two outbreaks. We also examined the distribution of durations of first outbreaks and calculated the 0.25, 0.50, 0.75, 0.90, and 0.95 quantiles of this distribution.

We then calculated targets describing infection dynamics from one week before the start of the first outbreak to one week after its conclusion. For each facility with an outbreak, we calculated the effective reproductive numbers (Rt) over this period using the R0 package and the Wallinga and Teunis method^40^ with correction for estimation in real time,^41^ using estimates of the generation time and its standard deviation from the literature.^29^^,^^30^^,^^36^, ^37^, ^38^, ^39^

Finally, we calculated targets describing cases and case rates. We computed mean cases per capita for facilities with an outbreak and without an outbreak. For those facilities with an outbreak, we computed mean cases per capita during the first outbreak. Finally, we computed the mean weekly case rate per capita before the first outbreak and after the first outbreak but before the second outbreak.

### Calibration

We calibrated a set of stochastic SEIDR compartmental models with one per ICE facility within the ICE system. The facilities had different population sizes based on empirical data.^27^ They also had different betas, which were governed by an ICE system-level gamma distribution. The facilities shared all other parameter values.

We employed IMIS calibration (Supplementary Methods, Text 1).^24^^,^^25^ In the initial stage, we selected very broad ranges for model parameters and generated uniform distributions (Supplementary Fig. S2). We sampled from these 50,000 times using orthogonal sampling.^42^ We ran the model 25 times per each parameter set and computed the model outputs analogous to each of the calibration targets we computed using the empirical data. We calculated the likelihood for each parameter set based on a joint likelihood function (Supplementary Methods, Text 2). To generate this joint likelihood function, we parameterized likelihood functions for each target separately and then multiplied them together. We conducted 10 importance sampling stages. In each stage, we sampled 2000 parameter sets according to the IMIS algorithm, ran the model 25 times for each of these parameter sets, and computed likelihoods. Finally, per the IMIS algorithm, we merged all parameter sets from all stages of IMIS and resampled 10,000 with replacement according to the IMIS weights. We used these parameter sets as inputs to our policy analyses.

### Policy analyses

We evaluated the effects that various levels of decarceration might have had on cumulative COVID-19 cases and infections among those incarcerated within the ICE system prior to the availability of vaccines, compared to the status quo (no substantial decarceration).

To project outcomes for the entire ICE system in the absence of substantial decarceration, we simulated outcomes for the ICE system once for each of our 10,000 parameter sets. We then calculated mean outcomes and associated uncertainties.

To model COVID-19 cases and infections under various levels of ICE system-level decarceration, we removed some fraction of people (10–90%) from all ICE facilities at the start of the simulation (May 6, 2020). The population in each facility was then maintained at this lower level throughout the simulation through December 31, 2020. Those removed were exposed to cumulative risks in the community during this period (described below). For those who remained incarcerated in the ICE facilities, we modeled changes to risks of transmission and infection under two potential assumptions about how decarceration would alter contact patterns. These included: 1) a reduction in contacts and hence a reduction in β that was proportional to the level of decarceration and 2) no reduction in contacts and hence no reduction in β.

Those who left ICE facilities no longer faced the facility-specific transmission and infection risks but instead faced analogous, but in general lower, risks in free-living communities during the May 6, 2020 to December 31, 2020 period of the pandemic. Because over 90% of people in ICE detention are of Hispanic ethnicity, we quantified the free-living community risks faced by Hispanic populations during this period.^43^ First, we used county-level data on cumulative detected cases and population sizes during this period from the CDC and U.S. Census^33^^,^^35^ to establish county-level cumulative risks for Hispanic adults. Next, using the county-level estimates from the covidestim model^34^ on overall (not only Hispanic) cumulative detected cases and infections during this period, we estimated the relationship between cumulative detected case risk and the cumulative infection risk using a general linear model with a log link. We used this relationship to predict the Hispanic-specific county-level cumulative infection risk based on the Hispanic-specific cumulative detected case risks we had previously computed. We then took the Hispanic-population-weighted average of these cumulative risks which we used for those released from ICE facilities into the free-living community. The covidestim model is no longer updated but still provides historical estimates.

See the supplement (Supplementary Methods, Text 3) for a more detailed discussion of model assumptions. See Supplementary Fig. S3 for a flowchart detailing the various steps in our analyses.

### Role of the funding source

The funders were not involved in the study design, data collection, analysis, interpretation of data, writing of the report, or the decision to submit the manuscript for publication.

## Results

Calibration results are shown in Supplementary Figs. S2, S4, S5. Simulation outputs from the calibrated models were concordant with the empirical calibration targets.

**Fig. 2:**
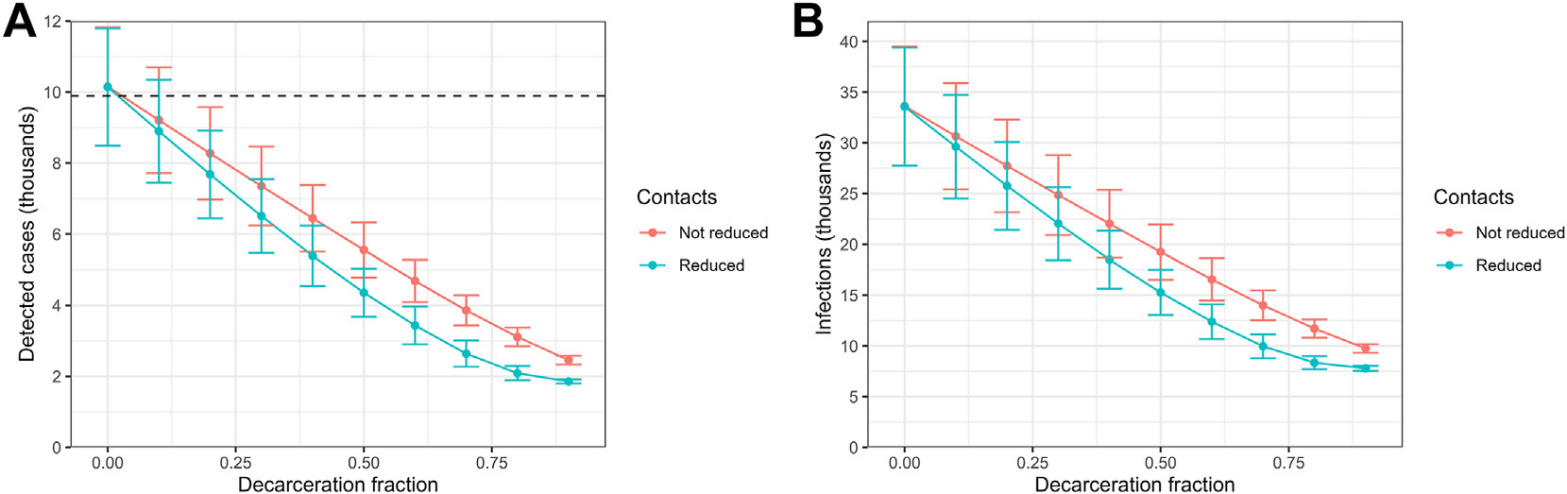
**Projected detected cases (A) and infections (B) at various levels of decarceration.** We considered decarceration fractions of 0.0–0.9 by increments of 0.1. Decarcerated people were exposed to community level risks of cases and infections. We modeled two scenarios following decarceration including 1) no reduction in contacts and 2) reduced contacts proportional to the fraction decarcerated. ICE system status quo projected cases were consistent with those empirically observed (dashed line).

Fig. 2 shows projected detected cases (Fig. 2a) and infections (Fig. 2b) at various levels of decarceration. With no decarceration, the ICE infection rate was 5.05 per 1000 person-days (95% CrI: 3.40–6.81) and case rate was 1.53 per 1000 person-days (95% CrI: 1.04–2.02). These were substantially higher than the community infection rate of 1.22 per 1000 person-days (95% CrI: 1.17–1.27) and case rate of 0.29 per 1000 person-days (95% CrI: 0.29–0.29).

With decarceration, the case and infection rates declined roughly proportional to the level of decarceration. For example, depending on assumptions about how contacts changed, a 30% reduction in the ICE population produced reductions in infection rates between 26% and 34%. The decline was approximately linear when decarceration did not reduce the number of contacts of people remaining in the facilities and faster than linear when it did reduce them. The former may occur, for example, when decarceration is implemented by closing select housing areas at facilities and operating the others at the same density. The latter may occur when decarceration is implemented by evenly removing people from the housing areas leaving the facilities operating at lower density. At every level of decarceration, cases and infections were substantially lower when contacts were reduced compared to when they were not reduced.

**Fig. 3:**
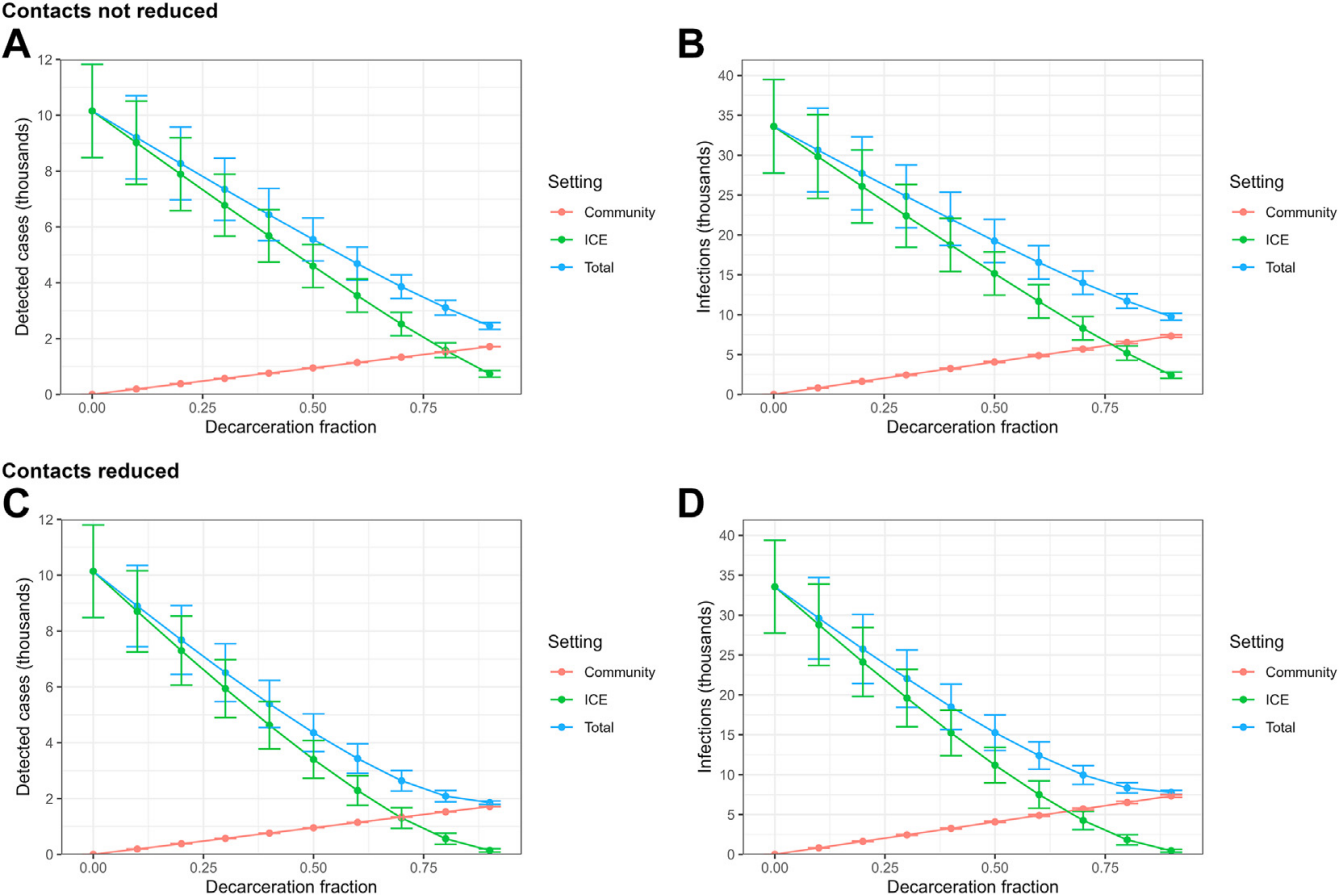
**Projected detected cases (A and C) and infections (B and D) at various levels of decarceration by origin of cases.** Plots show how the total cases and infections are composed of those that occur in the community and ICE settings.

Fig. 3 shows projected detected cases and infections at various levels of decarceration by whether the cases occurred in the ICE facilities or community. When contacts were not reduced, the total number of detected cases (Fig. 3a) and infections (Fig. 3b) fell approximately linearly as the level of decarceration increased. Considering these cases and infections by their origin, with decarceration, they rose in the community and fell in the ICE facilities as a higher fraction of the original population of people in ICE detention was moved to the community and exposed to community risks. However, at all decarceration levels we considered, community risks were still substantially lower than facility risks. Once approximately 75% of the incarcerated population in ICE facilities were decarcerated, cases and infections in facilities and the community were approximately equal.

When contacts were reduced, the total number of detected cases (Fig. 3c) and infections (Fig. 3d) fell faster than linearly as the level of decarceration increased. In this case, ICE cases and infections fell more precipitously such that once approximately 65% of the incarcerated population in ICE facilities were decarcerated, cases and infections in facilities and the community were approximately equal. At all decarceration levels we considered, except 90%, community risks were lower than facility risks (and even at 90%, they were comparable.)

## Discussion

By quantifying how many COVID-19 infections could have been averted through different decarceration strategies, we fill important knowledge gaps that can inform future health policy. During the pre-vaccine phase of the COVID-19 pandemic, people in ICE detention facilities faced COVID-19 case rates that were much higher than otherwise similar free-living people. ICE reported over 8500 COVID-19 cases detected among people detained in their facilities, representing nearly 10% of all people detained.^44^ The actual number of people infected was likely much higher, since testing was limited to those who were symptomatic and screened. In April 2020, ICE released operational guidance on mitigation strategies that included cleaning, disinfecting, and masking when personal protective equipment was available, and isolating symptomatic people, consistent with existing CDC guidance at the time.^45^ These strategies were unevenly and inconsistently implemented across ICE facilities, resulting in realization of a highly problematic scenario, in which tens of thousands of detained people became infected with COVID-19.^46^ Using simulation models calibrated to retrospective ICE data, we projected that many of these cases could have been avoided with decarceration and that even a modest reduction in facility populations would have had a meaningful impact on reducing COVID-19 cases and infections.

Our study lends further support to a growing literature, discussed earlier, suggesting that decarceration is a highly effective COVID-19 mitigation strategy for carceral settings. We add to this literature by examining the effects of decarceration, accounting for both level and implementation, on COVID-19 infections in the ICE system and the surrounding community. Our findings may have broader relevance beyond SARS-CoV-2 to other droplet- and air-borne infections, especially pathogens for which there is limited population immunity, either because no vaccine is readily available (e.g., emerging pathogens, multi-drug resistant organisms like tuberculosis^47^) or because the population has limited uptake of existing vaccines. In Massachusetts jails, for example, seasonal influenza vaccination rates prior to the COVID-19 pandemic ranged from 1.9 to 11.8%, which is far less than the national uptake of 30–60%.^48^ Influenza vaccination uptake is consistently lowest among people from minoritized racial/ethnic backgrounds,^49^ who are over-represented in carceral settings. In such settings with limited population immunity, our results suggest that decarceration can have a substantial impact on reducing new infections.

Our findings have important policy implications for pandemic preparedness and routine infection prevention. To be sure, public health is not the only lens through which decarceration can be viewed. Decarceration must also be considered in terms of medical-legal and human rights, particularly for people detained in ICE facilities. Decarceration also has important implications for immigration reform but these broader ethical and policy questions were beyond the scope of this analysis. From a pragmatic standpoint, we acknowledge that actual implementation of decarceration may be subject to local and national political will. In the absence of decarceration, there are other policies that can be implemented to enhance the health and well-being of incarcerated persons, even in overcrowded circumstances. These include limiting transfers, promoting personal hygiene, restricting visitors, trying to maximize space between people, and screening staff for symptoms. However, such guidelines were in place by both the CDC and ICE during our model period, suggesting that they may not be sufficient.^19^^,^^45^

Simulation models were designed to be as comprehensive as possible, though they were not without limitations. First, due to lack of data availability, we made a variety of simplifications while modeling such as not accounting for specific features of different facilities; as such, the models are not intended to make predictions about particular facilities, but to give a sense of what may be possible under reasonably conservative assumptions. Second, we did not directly account for transfers between facilities. However, we indirectly accounted for them by allowing infections to be introduced from external sources as described in the Methods section. Third, the models focused on the early period of the pandemic prior to the availability of vaccines; we did not explore how vaccine-induced immunity, long-term accumulation of natural immunity, or later more transmissible SARS-CoV-2 variants such as the Omicron variant, may have modified the health effects of decarceration. Fourth, as we only modeled cases and infections, we did not directly consider potential health outcomes of long COVID or other post-acute sequelae (such as cerebrovascular disease^50^ or new-onset diabetes^51^), though these would have likely increased the health benefits of decarceration. Fifth, we did not model how averting cases in the ICE system via decarceration might reduce community cases, potentially rendering our estimates of the health benefits of decarceration conservative. Sixth, in using a compartmental model, we assumed homogeneous mixing of people within each ICE facility. This is a common assumption when modeling infectious diseases in correctional settings due to high population density, shared areas, and staff. Seventh, we had to exclude some facilities with missing data; however, they represented a small fraction of the total facilities. Eighth, as we only modeled cases and infections, we did not consider cost savings associated with incarcerating fewer people following decarceration; however, this would have likely strengthened the argument for decarceration, given the very high societal expense of human incarceration. Policy makers for ICE facilities can easily assess these cost savings using their own internal data.

COVID-19 infection, especially in older and medically vulnerable adults, increases the risk of death or can contribute to overall mortality.^52^^,^^53^ We did not model deaths due to two limitations of the available data. First, there were concerns about it having systematic reporting inaccuracies.^54^ Second, we did not have access to data on individual-level factors, like age or comorbid conditions, to be able to accurately interpret such data. These data gaps reflect the importance of collecting and reporting this information routinely as other carceral systems, like California state prisons, feasibly did.^20^

Without substantial decarceration, people held in ICE detention facilities faced greatly elevated COVID-19 infection risks compared to free-living populations in the community. Our findings highlight the infection risks associated with incarceration, which compound other health harms of incarceration.

## Contributors

CW, JPM, DB, CB, FLA, and JG-F conceptualized the study. JPM helped acquire the data and CW and JG-F curated it. CW conducted the formal analysis, with supervision and oversight by JG-F. CW, JPM, JG-F, DB, and FLA interpreted the data. CW and JPM wrote the original draft of the manuscript with review and editing by CW, JPM, and JG-F. All authors had responsibility for the decision to submit the study for publication.

## Data sharing statement

We used publicly available datasets. Input data and statistical code for replication and extension of our analysis will be published at: https://purl.stanford.edu/hz524dq6106 concurrent with publication.

## Declaration of interests

None of the authors have competing conflicts of interest to declare.

## References

[1] Shoichet C. (2020). https://www.cnn.com/2020/03/20/health/doctors-ice-detention-coronavirus/index.html.

[2] Meyer J.P., Franco-Paredes C., Parmar P., Yasin F., Gartland M. (2020). COVID-19 and the coming epidemic in US immigration detention centres. Lancet Infect Dis.

[3] Saloner B., Parish K., Ward J.A., DiLaura G., Dolovich S. (2020). COVID-19 cases and deaths in federal and state prisons. JAMA.

[4] Global Detention Project (2020). https://www.globaldetentionproject.org/countries/americas/united-states.

[5] Widra E., Herring T. (2021). https://www.prisonpolicy.org/global/2021.html.

[6] Weyant C., Lee S., Andrews J.R., Alarid-Escudero F., Goldhaber-Fiebert J.D. (2023). Dynamics of respiratory infectious diseases in incarcerated and free-living populations: a simulation modeling study. Med Decis Making.

[7] Ward J.A., Parish K., DiLaura G., Dolovich S., Saloner B. (2021). COVID-19 cases among employees of U.S. Federal and state prisons. Am J Prev Med.

[8] Hooks G., Sawyer W. (2020). https://www.prisonpolicy.org/reports/covidspread.html#aggregate.

[9] Irvine M., Coombs D., Skarha J. (2020). Modeling COVID-19 and its impacts on U.S. Immigration and Customs enforcement (ICE) detention facilities, 2020. J Urban Health.

[10] National Academies of Sciences E, Medicine (2020).

[11] Malloy G.S.P., Puglisi L., Brandeau M.L., Harvey T.D., Wang E.A. (2021). Effectiveness of interventions to reduce COVID-19 transmission in a large urban jail: a model-based analysis. BMJ Open.

[12] Vest N., Johnson O., Nowotny K., Brinkley-Rubinstein L. (2021). Prison population reductions and COVID-19: a latent profile analysis synthesizing recent evidence from the Texas state prison system. J Urban Health.

[13] Jiménez M.C., Cowger T.L., Simon L.E., Behn M., Cassarino N., Bassett M.T. (2020). Epidemiology of COVID-19 among incarcerated individuals and staff in Massachusetts jails and prisons. JAMA Netw Open.

[14] Lofgren E.T., Lum K., Horowitz A., Mabubuonwu B., Meyers K., Fefferman N.H. (2022). Carceral amplification of COVID-19: impacts for community, corrections officer, and incarcerated population risks. Epidemiology.

[15] Towers S., Wallace D., Walker J., Eason J.M., Nelson J.R., Grubesic T.H. (2022). A study of SARS-COV-2 outbreaks in US federal prisons: the linkage between staff, incarcerated populations, and community transmission. BMC Publ Health.

[16] Servick K. (2020). Pandemic inspires push to shrink jails, prisons. Science.

[17] Reinhart E., Chen D.L. (2021). Association of jail decarceration and anticontagion policies with COVID-19 case growth rates in US counties. JAMA Netw Open.

[18] Interagency Standing Committee (2020). https://www.who.int/publications/m/item/covid-19-focus-on-persons-deprived-of-their-liberty-(interim-guidance-jointly-developed-by-iasc-ohchr-who).

[19] Centers for Disease Control and Prevention (2020). https://www.cdc.gov/coronavirus/2019-ncov/community/correction-detention/guidance-correctional-detention.html.

[20] Chin E.T., Ryckman T., Prince L. (2021). COVID-19 in the California state prison system: an observational study of decarceration, ongoing risks, and risk factors. J Gen Intern Med.

[21] National Immigration Forum Fact sheet: immigration detention in the United States. https://immigrationforum.org/wp-content/uploads/2021/01/Immigration-Detention-Factsheet_FINAL.pdf.

[22] Vera ICE detention trends. https://www.vera.org/ice-detention-trends.

[23] Keeling M., Rohani P. (2008).

[24] Raftery A.E., Bao L. (2010). Estimating and projecting trends in HIV/AIDS generalized epidemics using incremental mixture importance sampling. Biometrics.

[25] Steele R.J., Raftery A.E., Emond M.J. (2006). Computing normalizing constants for finite mixture models via incremental mixture importance sampling (IMIS). J Comput Graph Stat.

[26] (2023). Project; TCP. COVID prison project data.

[27] TRAC Immigration (2023). https://trac.syr.edu/immigration/detentionstats/facilities.html.

[28] R Core Team (2020).

[29] Alarid-Escudero F., Gracia V., Luviano A. (2021). Dependence of COVID-19 policies on end-of-year holiday contacts in Mexico City metropolitan area: a modeling study. MDM Policy Pract.

[30] Ryckman T., Chin E.T., Prince L. (2021). Outbreaks of COVID-19 variants in US prisons: a mathematical modelling analysis of vaccination and reopening policies. Lancet Public Health.

[31] Goldhaber-Fiebert J.A.-E.F., Andrews J. (2020). https://www.sc-cosmo.org/wp-content/uploads/2020/11/SC_COSMO_TechnicalDescription_v02.pdf.

[32] Gillespie D.T. (2001). Approximate accelerated stochastic simulation of chemically reacting systems. J Chem Phys.

[33] Centers for Disease Control and Prevention (2023). https://data.cdc.gov/Case-Surveillance/COVID-19-Case-Surveillance-Public-Use-Data-with-Ge/n8mc-b4w4.

[34] covidestim (2023). https://covidestim.org/.

[35] U.S. Census Bureau Population Division (2020). https://www2.census.gov/programs-surveys/popest/datasets/2010-2019/counties/asrh/cc-est2019-alldata.csv.

[36] Lauer S.A., Grantz K.H., Bi Q. (2020). The incubation period of coronavirus disease 2019 (COVID-19) from publicly reported confirmed cases: estimation and application. Ann Intern Med.

[37] He X., Lau E.H.Y., Wu P. (2020). Temporal dynamics in viral shedding and transmissibility of COVID-19. Nat Med.

[38] Ashcroft P., Huisman J.S., Lehtinen S. (2020). COVID-19 infectivity profile correction. Swiss Med Wkly.

[39] Li Q., Guan X., Wu P. (2020). Early transmission dynamics in wuhan, China, of novel coronavirus-infected pneumonia. N Engl J Med.

[40] Wallinga J., Teunis P. (2004). Different epidemic curves for severe acute respiratory syndrome reveal similar impacts of control measures. Am J Epidemiol.

[41] Cauchemez S., Boëlle P.Y., Thomas G., Valleron A.J. (2006). Estimating in real time the efficacy of measures to control emerging communicable diseases. Am J Epidemiol.

[42] Owen A. (1992). Orthogonal arrays for computer experiments, integration and visualization. Stat Sin.

[43] Ryo E., Peacock I. (2018).

[44] United States Government Accountability Office (2021).

[45] US Immigration and Customs Enforcement EaRO (2020). https://www.ice.gov/doclib/coronavirus/eroCOVID19responseReqsCleanFacilities-v1.pdf.

[46] Niu I., Rhyne E. (2021). https://www.nytimes.com/2021/04/25/video/immigration-detention-covid-takeaways.html.

[47] Stuckler D., Basu S., McKee M., King L. (2008). Mass incarceration can explain population increases in TB and multidrug-resistant TB in European and central Asian countries. Proc Natl Acad Sci USA.

[48] Khorasani S., Zubiago J., Carreiro J., Guardado R., Wurcel A.G. (2022). Influenza vaccination in Massachusetts jails: a mixed-methods analysis. Public Health Rep.

[49] McGovern I., Bogdanov A., Cappell K., Whipple S., Haag M. (2022). Influenza vaccine uptake in the United States before and during the COVID-19 pandemic. Vaccines (Basel).

[50] Wang W., Wang C.Y., Wang S.I., Wei J.C. (2022). Long-term cardiovascular outcomes in COVID-19 survivors among non-vaccinated population: a retrospective cohort study from the TriNetX US collaborative networks. EClinicalMedicine.

[51] Rahmati M., Yon D.K., Lee S.W. (2023). New-onset type 1 diabetes in children and adolescents as postacute sequelae of SARS-CoV-2 infection: a systematic review and meta-analysis of cohort studies. J Med Virol.

[52] Fernández Villalobos NV., Ott J.J., Klett-Tammen C.J. (2021). Effect modification of the association between comorbidities and severe course of COVID-19 disease by age of study participants: a systematic review and meta-analysis. Syst Rev.

[53] Centers for Disease Control and Prevention (2022). https://www.cdc.gov/coronavirus/2019-ncov/science/data-review/primary-cause.html.

[54] Turcotte M., Sherman R., Griesbach R., Hinga Klein A. (2021). https://www.nytimes.com/2021/07/07/us/inmates-incarcerated-covid-deaths.html.

