# Supplementary material for: Decarceration and COVID-19 infections in U.S. Immigration and Customs Enforcement detention facilities: a simulation modeling study
Source: Lancet Reg Health Am. 2024 Dec 27;42:100971. doi: 10.1016/j.lana.2024.100971 (PMC11741939; doi:10.1016/j.lana.2024.100971)
Supplement: Supplementary Methods and Figs. S1–S5 [file mmc1.docx]

**Decarceration and COVID-19 Infections in U.S. Immigration and Customs Enforcement Detention Facilities: A Simulation Modeling Study**

Supplement: Table of Contents

[Supplementary Methods 2](#_Toc182580654)

[Supplementary Figure 1. Selection of ICE facilities for analyses 6](#_Toc182580655)

[Supplementary Figure 2. Prior and posterior distributions for calibrated parameters 7](#_Toc182580656)

[Supplementary Figure 3. Flowchart detailing the various steps in our analyses 8](#_Toc182580657)

[Supplementary Figure 4. Goodness of fit of calibrated parameter sets 9](#_Toc182580658)

[Supplementary Figure 5. Incidence of detected cases over time in each ICE facility according to our model 10](#_Toc182580659)

# **Supplementary Methods**

*Text 1. Overview of IMIS.*

We calibrated our model using IMIS.^1,2^ In the initial stage of IMIS, N_0_ parameter sets are sampled from a prior distribution. These parameter sets are run through the model and likelihoods are computed for each parameter set based on the correspondence between model outputs for that parameter set and the empirical targets. The IMIS weights for each parameter set in the first stage are calculated by dividing the likelihood of each parameter set by the sum of the likelihoods for all parameter sets. In the k importance sampling stages, a multivariate Gaussian distribution is parameterized. This distribution is centered at the maximum weight parameter set (θ^(k)^) and has covariance equal to the weighted covariance of the B inputs that are closest to θ^(k)^ in terms of the Mahalanobis distance. B new parameter sets are then sampled from this distribution. They are run through the model and likelihoods are computed. New weights are computed, which are functions of the likelihood, prior distribution, and mixture sampling distribution. Finally, after the importance sampling stages, the parameter sets from all stages are combined and J parameter sets are sampled with replacement according to the weights. The algorithm is unbiased for any choice of these control parameters since it is based on importance sampling.

*Text 2. Joint likelihood function.*

We determined target means and standard deviations by bootstrapping. We then parameterized a distribution for each target. For targets that were fractions, we used beta distributions. For targets between zero and positive infinity, we used gamma distributions. Finally, for the outbreak durations (discrete and in weeks), we used the bootstrapped probability mass functions. We computed a joint likelihood function by multiplying together the separate likelihood functions for each target.

We recognize that we were unable to model all sources of uncertainty. For example, there could have been inaccurate reporting or inaccurate testing. As such, we increased the standard deviation of all target distributions by 50% before computing likelihoods. For the outbreak durations (discrete and in weeks), we added 0.01 probability to each valid outbreak duration up to the max duration in the empirical data (7 weeks) and then renormalized so the probabilities summed to 1. Slightly expanding target distributions in this way to account for other sources of uncertainty is standard practice in calibration of health policy models. Furthermore, our model output distributions still show good concordance with the unwidened target distributions (Figure S4).

*Text 3. More detailed discussion of model assumptions.*

In our use of an SEIDR compartmental model, we assume homogeneous mixing of people within each ICE facility. This is a reasonable assumption as these settings have high connectivity due to factors such as high population density, shared areas, and staff. People are not just isolated in single cells all day. The high Rt in these settings also makes homogeneous mixing a reasonable assumption. This assumption is common in the literature and compartmental models have been widely used to model infectious diseases including COVID-19 in prisons and jails.

As described in the methods section, we either based parameter values on published sources or calibrated them. No parameter values were assumed.

We did not consider the birth rate or natural death rate as they would be negligible over our relatively short model period of 240 days. This is common practice for such short-term models.

We modeled a constant infection introduction rate even though it may vary over time and between facilities. We believe this is a reasonable approximation as we just model cumulative outcomes, and the facilities had similar numbers of outbreaks in the empirical data suggesting they had similar infection introduction rates.

We had different criteria for when facilities implemented mitigation strategies versus had an outbreak. We assumed that an ICE facility would implement mitigation efforts with some delay after it detects its first case. We assumed this as a case in an ICE facility would likely be a major concern and officials would likely try to respond appropriately. However, there would likely be some delay as it takes time for officials to agree upon a course of action, gather necessary resources, and implement it. In contrast, outbreaks were defined for the purposes of calibration. Empirically, ICE facilities experienced occasional cases but also sometimes had larger outbreaks (i.e., periods with many more cases within a relatively short time). For example, if a facility had two major outbreaks, it is important to be able to approximately reproduce this outcome with the model. Per the methods section, we defined an outbreak as over 2% of the population having detected cases each week for any contiguous period of at least 3 weeks. We selected this definition as it means the facility had a substantial, sustained outbreak. However, in principle, other reasonable definitions of an outbreak could have been used, with the key piece being that the definition was used both to characterize the empirical data forming the calibration targets as well as the simulation results pertaining to outbreaks used to calibrate the model parameters.

We assumed that mitigation effort intensity was reduced to baseline levels after 2 weeks with no detected cases at the facility; mitigation could be reactivated if subsequent cases were detected. We made this assumption as facilities would be unlikely to leave mitigation strategies in place indefinitely and 2 weeks is much longer than the sum of the expected durations in the E and I states. This assumption may, however, oversimplify the true dynamics of policy implementation and adherence. Our code will be publicly available to allow others to explore alternative assumptions.

We calculated targets describing infection dynamics from one week before the start of the first outbreak to one week after its conclusion. We considered this period as infections were likely abnormally high before and after outbreaks, and this data might help inform estimation of the effective reproductive numbers (Rt).

Finally, in IMIS, we selected control parameters that gave us good efficiency and based on our computational constraints. The IMIS algorithm is unbiased for any choice of these control parameters since it is based on importance sampling.

*References*

1 Raftery AE, Bao L. Estimating and Projecting Trends in HIV/AIDS Generalized Epidemics Using Incremental Mixture Importance Sampling. *Biometrics.* 2010;66:1162–73.

2 Steele RJ, Raftery AE, Emond MJ. Computing Normalizing Constants for Finite Mixture Models via Incremental Mixture Importance Sampling (IMIS). *Journal of Computational and Graphical Statistics.* 2006;15:712–34.

# **Supplementary Figure 1. Selection of ICE facilities for analyses.**

ICE facilities with case data

(n = 126)

Screened for those with population data (n = 109)

Screened for those with mean population ≥ 20 (n = 95)

Screened for those with ≥ 200 days of data (n = 78)

Screened for those in 50 US states and DC (n = 108)

17 facilities excluded

1 facility excluded

13 facilities excluded

17 facilities excluded

# **Supplementary Figure 2. Prior and posterior distributions for calibrated parameters.**

# **Supplementary Figure 3. Flowchart detailing the various steps in our analyses.**

We developed a set of stochastic SEIDR simulation models of SARS-CoV-2 transmission in ICE facilities.

We calibrated model parameters to empirical data using the IMIS algorithm. This yielded 10,000 parameter sets, which formed our posterior distributions.

For no decarceration, we ran the simulation models once for each of the 10,000 parameter sets. We calculated various outcomes and compared them to the empirical data.

For each decarceration scenario, we ran the simulation models once for each of the 10,000 parameter sets. We calculated various outcomes.

# **Supplementary Figure 4. Goodness of fit of calibrated parameter sets.**

We show the distributions of model outcomes versus those for the targets.

# **Supplementary Figure 5. Incidence of detected cases over time in each ICE facility according to our model.**

We simulated incidence of detected cases over time for each facility once using the highest likelihood parameter set from IMIS. Each facility is represented by one line and is shown in its own plot.
